# The Use of Artemether-Lumefantrine for the Treatment of Uncomplicated *Plasmodium vivax* Malaria

**DOI:** 10.1371/journal.pntd.0001325

**Published:** 2011-12-27

**Authors:** Quique Bassat

**Affiliations:** Barcelona Centre for International Health Research (CRESIB), Hospital Clínic Universitat de Barcelona, Barcelona, Spain; The George Washington University Medical Center, United States of America

## Abstract

The long-standing dearth of knowledge surrounding *Plasmodium vivax*, the most widely distributed of the malaria species, merits urgent attention. A growing awareness of the true burden of this parasite and its potential to cause severe disease, and the identification of increasing parasite resistance in many areas of the world to chloroquine, the mainstay of vivax treatment, underscores the need to identify new and effective treatment strategies. Artemisinin-based combination therapies (ACTs) have been widely adopted as first-line treatment for *P. falciparum* malaria and would offer logistic benefits in areas of co-endemicity. However, while ACTs show high and similar efficacy against the blood stages of *P. vivax*, neither ACTs nor chloroquine are active against vivax hypnozoites and must be complemented with a full course of primaquine to eradicate dormant vivax hypnozoites and prevent relapses. Artemether-lumefantrine (AL), the most commonly deployed ACT, has shown rapid clearance of *P. vivax* parasitemia and fever. The relatively short half-life of lumefantrine would appear beneficial in terms of reducing risk of resistance when compared to other ACTs. However, it has a shorter capability to suppress vivax relapses or prevent de novo infections, which generally translates into comparatively lower in vivo short-term measures of efficacy (e.g., day 28 or day 42 uncorrected cure rates). Assuming that the different artemisinin derivatives have equivalent efficacy against vivax, differences between AL and other ACTs may be restricted to the duration of plasma therapeutic levels of the partner drug, a variable of limited clinical relevance, particularly in regions with low vivax transmission rates or in cases where primaquine is added to the regimen to prevent relapses. More rigorous assessment of the use of ACTs in general, and AL in particular, for the treatment of *P. vivax* infections, either alone or in combination with primaquine, is merited. In the meantime, AL treatment of vivax malaria may be a pragmatic choice for areas with chloroquine-resistant *P. vivax*, and in co-endemic areas where AL is already used routinely against *P. falciparum* and parasitological differentiation is not routinely performed or only clinical diagnosis is used.

## Introduction

*Plasmodium vivax* infection persists as a major global health problem. It is more widely distributed than *P. falciparum* [1], with over 2.5 billion people living at risk and an estimated 80 to 300 million clinical cases each year [2], [3]. It is common in Asia, Oceania, Central and South America and the Middle East [1], [3], but its burden is also increasingly recognized in East Africa [4]–[6] and, more recently, in other African regions with reports from western African countries including Democratic Republic of the Congo, Côte D'Ivoire, and Equatorial Guinea [1], [7]. The belief that the virtual absence of the red blood cell Duffy-positive phenotype among black Africans protects these populations against *P. vivax* infections [8] (the Duffy protein is thought to be necessary for the parasite's invasion of reticulocytes) is under reconsideration [9], and it is now hypothesized that *P. vivax* may invade young red blood cells using alternative mechanisms [10].

Traditionally, *P. vivax* has been regarded as a benign infection. This idea, however, has recently been challenged [2], [11]–[14], and the literature reflects increasing reports of vivax-attributable severe or even life-threatening illness [15]–[17]. Episodes of *P. vivax* infection should thus be regarded as potentially lethal and should prompt urgent treatment with effective antimalarial medication.

In the majority of settings, *P. vivax* coexists with *P. falciparum* [18]–[20]. Although the examination of thin blood slides using optical microscopy has traditionally been used for species differentiation, accurate species diagnosis is difficult, and ultimately may require highly specific PCR methods, not applicable in the daily clinical routine. The sensitivity of rapid diagnostic tests is limited when trying to differentiate between these two species, especially at low parasitemias, common in *P. vivax* infections [21], [22]. In resource-challenged regions, empiric treatment based on clinical suspicion is widely accepted and implemented, although no longer recommended by the World Health Organization (WHO) [20].

Programmatically, the use of a single first-line therapy effective against both *P. vivax* and *P. falciparum* would be ideal in view of the frequent co-endemicity of the two species and the increasing resistance of parasites to chloroquine. The widespread adoption of artemisinin-based combination therapies (ACTs) as highly effective first-line therapy for *P. falciparum* has prompted a closer examination of their role in the management of *P. vivax* malaria. This article considers the available evidence relating to the potential role of artemether-lumefantrine (AL), the most widely used ACT worldwide, in the management of vivax malaria.

## Methods

An electronic search of articles published on or before 31 January 2011 was performed in EMBASE, Medline, PubMed, and BIOSIS Previews. Search terms were “artemether-lumefantrine,” “artemether,” “artemisinin,” “*Plasmodium vivax*,” and “vivax,” with no time or language restrictions. Search results were examined for clinical studies of AL in the treatment of uncomplicated *P. vivax* malaria, including subpopulation analyses. The limited number of relevant, well-designed trials meant that no meaningful meta-analysis could be performed.

## *P. vivax* Life Cycle and Implications for the Evaluation of Efficacy of Antimalarials

**Figure 1:**
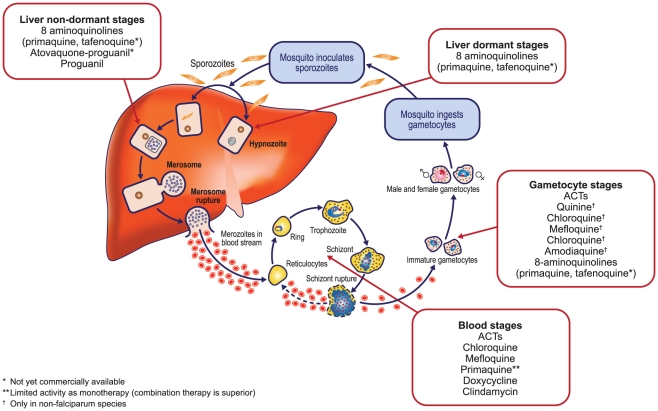
*P. vivax* life cycle and sites of action for different antimalarials.

In contrast to *P. falciparum*, *P. vivax* forms hypnozoites that can remain dormant in the parenchymal cells of the host liver following an acute infection. After an interval of time, which varies in duration depending on the geographical area [3], the hypnozoites can mature into hepatic schizonts that rupture to release merozoites capable of infecting erythrocytes and inducing a spontaneous relapse (Figure 1). Clinically, relapses present as a new malaria episode, indistinguishable from a new infection.

Thus, in *P. vivax* infections, the eradication of blood schizonts is not sufficient to control the disease, and an effective treatment requires killing the hypnozoites (“radical cure”) to prevent future relapses. Chloroquine, an inexpensive and effective treatment for vivax malaria in most areas of the world [23] has been the mainstay first-line therapy for this species for the past seven decades. It is still WHO's recommended drug for vivax, but needs to be combined with primaquine, currently the only approved drug capable of achieving radical cure of hypnozoites [18], [24], [25]. WHO guidelines state that primaquine needs to be administered daily for 14 d to achieve this purpose [20], although the efficacy of shorter (7 d) courses is being investigated. Chloroquine achieves parasitological cure at day 28 in more than 90% of chloroquine-sensitive *P. vivax* infections [26], but in many areas of the world, significant levels of resistance to this drug in *P. vivax* have been documented [19], [27], [28], notably in Indonesia (<50% probability of therapeutic success) [19] and to a lesser extent in India [29], Myanmar [30], Turkey [31], the Brazilian Amazon [32], and Colombia [33]. Worryingly, there is also growing evidence of clinical resistance of vivax to chloroquine in Africa [34]–[36]. Emergence of resistance to chloroquine, particularly in view of the potentially serious consequences of vivax infection, adds a further urgency to providing effective therapy.

**Figure 2:**
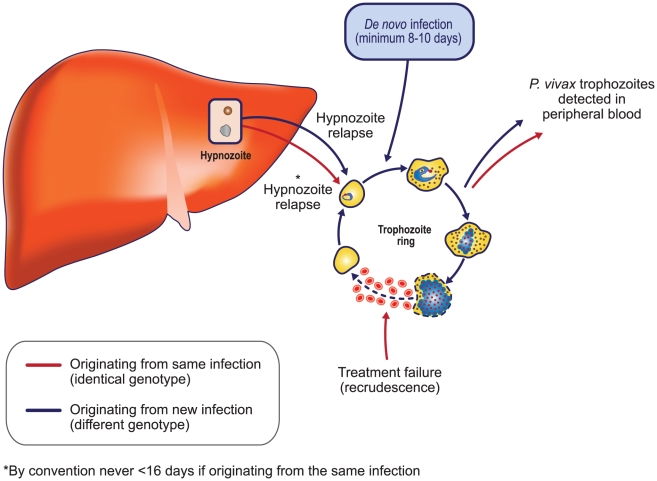
Possible sources of residual blood parasitemia after initial treatment and possible genetic similarity or dissimiliarity.

The possibility of relapse brings about uncertainty when assessing the efficacy of antimalarial drugs that have an effect on asexual stages of the life cycle but not on the dormant liver forms. Thus, patients correctly treated with an antimalarial with no antihypnozoite activity may present with recurrent post-treatment parasitemia that can derive from one of three sources: (1) reappearing parasites as a result of the incomplete clearance of the original infection (recrudescence), often the consequence of ineffective or incomplete treatment, (2) generation of de novo parasitemia from the liver reservoirs (relapse), or (3) parasites ensuing from a new and independent infection (new infection) (Figure 2). Parasites in recrudescent or relapsing infections may be genetically identical to the original infection and thus impossible to differentiate with current technology. Some reports state that over half of the relapsing parasites may be genetically different from the preceding documented infection, but this may be explained by older heterologous hypnozoites becoming reactivated [37]. A new infection arising from the bite of an infected vector, however, may differ from the original infecting parasite such that they can be distinguished from one another by PCR molecular techniques. A pragmatic solution to this investigative hurdle has been adopted whereby any new parasitemia appearing before day 16 is by convention directly classified as recrudescence (i.e., treatment failure) because of the unlikelihood of the infection being a relapse within such a short space of time, and because this is the minimum incubation period for a new infection to appear in peripheral blood. Reappearance of parasites after day 16 cannot be assumed to be recrudescence [20].

## ACTs in Vivax Malaria

ACTs are highly effective against uncomplicated *P. falciparum* malaria and are now recommended as first-line therapy [20], having been adopted in most countries where *P. falciparum* is endemic. Most ACTs show high and similar efficacy against the blood stages of *P. vivax* [38], but none is active against the hepatic *P. vivax* hypnozoites responsible for relapse [39]. Therefore, in order to achieve radical cure, similar to chloroquine treatment, ACT treatment must be complemented with a full course (14 d) of primaquine.

Where both *P. vivax* and *P. falciparum* species are prevalent and ACTs have been adopted as first-line therapy for *P. falciparum*, use of a unified first-line therapy based on ACTs would simplify treatment procurement, distribution, and management, offering interesting logistical benefits. ACT therapy for both types of infections would also avoid the problem of *P. falciparum* being mistakenly diagnosed as *P. vivax* and inadequately treated with chloroquine in the many regions where diagnosis is based on clinical symptoms alone [18]. In areas of chloroquine-resistant *P. vivax*, and where ACTs have already been adopted for the treatment of *P. falciparum* malaria, ACTs could also be used for the treatment of vivax, provided they are complemented with the standard primaquine course [20]. Indeed, at least four countries have now adopted a unified first-line therapy based on ACTs: the Solomon Islands, Vanuatu, and Papua New Guinea deploy AL, while Indonesia deploys dihydroartemisinin-piperaquine (DHA-PQP) in Papua [18].

## Rationale for Use of AL

A large evidence base is now available to demonstrate the efficacy of AL in the treatment of uncomplicated malaria in a range of patient types and locations worldwide, but particularly for *P. falciparum* infections. This includes the most extensive data for any ACT in children and pregnant women [40]–[44], the two populations most vulnerable to malaria. AL also offers the most extensive safety data base of all ACTs [45], being the most widely used ACT for falciparum malaria worldwide, with over 400 million treatments having already been distributed. Moreover, it is the only ACT with a formulation specifically tailored to use in children that is recommended by WHO. Last, but not least, resistance to lumefantrine in field isolates has not yet been convincingly demonstrated, an important advantage attributed to the relatively short half-life of lumefantrine [46] and the fact that, in contrast to most other ACT partner drugs, lumefantrine has never been used as monotherapy.

## Impact of the Partner Drug's Half-Life on Efficacy of ACTs in Vivax Infections

ACTs in which the partner drug has a long half-life are theoretically considered preferable in vivax infections [20], since this would be expected to extend the period of prophylaxis against either new infections or latent ones ensuing from the dormant hypnozoites in the liver. In an analysis of 10,549 patients treated for falciparum malaria on the Thailand–Myanmar border during 1991–2005, 11% (*n* = 1,164) of whom had mixed falciparum/vivax infection at screening, the rate of *P. vivax* infection by day 63 was 12.0% (*n* = 1,269) for vivax monoinfection and 0.8% (*n* = 86) for mixed infections [47]. ACTs that included partner drugs with shorter half-lives increased the risk of vivax infection recurrence by day 63 after treatment for *P. falciparum* malaria: the more rapidly eliminated AL and artesunate-atovaquone-proguanil combinations were associated with a 3.6-fold and 4.2-fold higher adjusted hazard ratio, respectively, for *P. vivax* infection than artesunate-mefloquine (*p*<0.001). No difference was observed for AL versus DHA-PQP or artemether-mefloquine combinations.

Lumefantrine, the partner drug in AL, has a relatively short half-life (3–6 d [48]) compared to mefloquine 12–15 d [49], amodiaquine 1–3 wk [50], or sulfadoxine 6–11 d [51]. Pyrimethamine and lumefantrine share a similar half-life (2–3 d [52]). Other than for chloroquine (up to 2 mo [50]) and piperaquine (23–28 d [53]), the differences in half-lives are, however, relatively small, and the advantage of a longer half-life is potentially counterbalanced by an increased risk for selection of resistant parasites, especially in areas of intense transmission [46], [54].

Antimalarial drugs have the potential to suppress the relapse of hepatic dormant stages as long as plasmatic levels remain above the therapeutic threshold. However, when drug concentrations decrease or even disappear, so does the capacity of the drug to inhibit relapses. Thus, although artemether individually shows similar efficacy rates to artesunate [38], standard measures of efficacy (e.g., 28-d parasitemia cure rate) are generally lower with AL than with other ACTs because lumefantrine is cleared from the blood rather quickly, leading to potentially earlier relapses. However, relapse of vivax infection is only delayed, and not prevented, by longer-acting partner drugs since an extended half-life does not influence the viability of hypnozoites in the liver and relapse can still occur once the drug is cleared. Over longer follow-up periods, the incidence of relapse would be expected to converge regardless of which ACT was employed. It has been proposed that an accurate comparison of relapse rates in vivax malaria requires at least 2 mo of follow-up [55], which is only rarely undertaken in clinical studies.

## Efficacy of AL in *P. vivax* Infection

Clinical investigations into the efficacy of antimalarial agents have been far less extensive for *P. vivax* than for *P. falciparum*, confirming its relative neglect [13]. Indeed, only a handful of clinical trials of AL in the treatment of *P. vivax* monoinfection have been published [34], [38], [56], [57]. In most studies [58]–[68], data on AL efficacy have been derived from subpopulation analyses within larger studies of both *P. falciparum* and *P. vivax* infections.

## Clinical Response

AL is highly effective against the blood stages of *P. vivax* infection, consistent with results from trials of AL in falciparum malaria [42], [69], [70]. The most informative indicator of efficacy is parasitological cure, since the clinical failure rate excludes asymptomatic parasitological failures that are detected only at routine follow-up visits. Nevertheless, it is important to note that fever resolution is rapid following AL therapy [38], [56], [57], [59].

Parasite clearance [38], [56], [57] is achieved rapidly in vivax-infected patients. This is not unexpected, since in vitro data have shown similar efficacy for artemether versus asexual forms of *P. falciparum* and *P. vivax* [44]. Reports from subpopulations with vivax monoinfection [38], [56], [57], [59] or mixed vivax and falciparum infection [57], [59], [60], [65], [67] have shown rapid clearance of both species' parasitemia in all patients following AL treatment (Table 1). In a cohort of 33 children with vivax monoinfection, mean time to parasite clearance following AL treatment was 33.6 h, with all patients being fully cleared of their parasites by day 7 [59].

**Table 1:** Parasite and fever clearance times in patients treated with artemether or AL for *P. vivax*.

Study	Population (Location)	Treatment	Sample Size	Parasite Clearance Time		Fever Clearance Time	
				Mean Time (Hours)	*p*-Value	Mean Time (Hours)	*p*-Value
Karunajeewa et al. [59]	Children 0.5–5 y (Papua New Guinea)	AL	33	33.6	<0.001	50.4	0.94
		Chloroquine-sulfadoxine-pyrimethamine	51	74.4		55.2	
		Artesunate-sulfadoxine-pyrimethamine	39	26.4		50.4	
		DHA-PQP	38	28.8		45.6	
Krudsood et al. [57]	Adults (≥15 y) (Indonesia)	AL-primaquine	47	41.6 (mean; range 14–71)	<0.001	21.8 (mean; range 4–70)	0.12
		Chloroquine- primaquine	51	55.8 (mean; range 23–106)		25.3 (mean; range 4–90)	
Li et al. [56]	Age not specified (China)	AL^a^	36	33.5	<0.01	22.3 (mean)	n.s.
		Chloroquine- primaquine	55	44.9		25.0 (mean)	
Pukrittayakamee et al. [38]	Adults (≥15 y) (Thailand)^b^	Artemether^c^	20	50	n.s. for artemether versus chloroquine-primaquine; *p* = 0.02 for artemether versus artesunate	14	<0.001 for artemether or artesunate versuss other treatments
		Artesunate	20	38		17	
		Chloroquine-primaquine	30	64		30	
		Chloroquine	30	65		31	
		Primaquine	30	83		28	
		Quinine	22	98		31	
		Mefloquine	20	76		21	
		Halofantrine	23	85		21	
		Pyrimethamine-sulfadoxine	12	114		58	

a Standard 3-d AL regimen.

b Data on parasite clearance time available for 195/207 patients.

c 2.7 mg/kg on day 1, 1.3 mg/kg/d for a further 4 d.

n.s., not significant.

### Comparison with Chloroquine and Other Antimalarials

One randomized trial compared the therapeutic response against vivax monoinfection of different orally administered antimalarials in a Thai population of 207 patients [38]. The rate of parasite clearance was markedly faster with artemether and artesunate than with the other non-ACT antimalarials (Table 1). When the baseline parasite count was taken into consideration, by calculating the ratio of the parasite count before treatment to the count at 48 h (“parasite reduction rate”), clearance rates were higher with artemether (median 1,720), with values 14-fold greater than for the other treatments, with the exception of artesunate (median 1,507). Correspondingly, the mean fever clearance time (defined as the time for body temperature to fall below 37.5°C and remain below this value for >48 h) was fastest in patients treated with artemether or artesunate compared to other antimalarials in this study [38], consistent with results from a randomized trial in Indonesia [56] (Table 1). Other authors have reported a similar time to resolution of fever with AL or chloroquine-primaquine [56].

## *P. vivax* Relapse

### Comparison with Chloroquine

As discussed above, the relatively short lumefantrine half-life often implies a lower day 28 uncorrected parasitological cure rate with AL than with antimalarials that have a longer half-life, including chloroquine and chloroquine-primaquine [34], [71]. In a recent study of AL versus chloroquine monotherapy in 133 patients with vivax malaria in Ethiopia, the uncorrected day 28 failure rate for AL was 19.0% (95% CI 2.9%–18.9%), compared to 7.5% for chloroquine (95% CI 11%–31.6%) [34] (Table 2). This difference would be expected in view of the relatively long half-life of chloroquine [50]. Resistance to chloroquine and subtherapeutic drug levels could explain the five recrudescences observed in the chloroquine group, but amongst the 19 cases of treatment failure in AL-treated patients [34], it is not possible to differentiate between relapse, recrudescence, or reinfection. One could speculate that most new parasitemias probably derived from relapse rather than recrudescence, since all but one treatment failure occurred during days 21–28; new infections were also possible, but unlikely because of the low transmission intensity in the area. The authors also suggested that since the evening doses of AL were unsupervised, full compliance could not be confirmed, although good compliance with the AL dosing regimen has generally been observed [72], [73].

**Table 2:** Parasitological failure in patients treated with AL for *P. vivax*.

Study	Population (Location)	Follow-Up	Treatment	Sample Size	Parasitological Failure	*p*-Value
Yohannes et al. [34]	Adults and children ≥1 y (Ethiopia)	28 d	AL	75	19%	0.015
			Chloroquine	87	7.5%	
Li et al. [56]	Age not specified (China)	9 mo	AL	36	84.9%^a^	<0.01
			Chloroquine- primaquine	55	22.9%^a^	
Pukrittayakamee et al. [71]	Adults (≥15 y) (Thailand)	28 d	Artemether^b^	20	52.9%^c^	—
			Artesunate	20	63.2%^c^	
			Chloroquine-primaquine	26	0^c^	
			Chloroquine	49	0^c^	
			Primaquine	30	11.5%^c^	
			Quinine	22	64.7%^c^	
			Mefloquine	16	0^c^	
			Halofantrine	23	52.9%^c^	
Karunajeewa et al. [59]	Children 0.5–5 y (Papua New Guinea)	28 d^d^	AL	33	45.5%	0.001 for AL versus DHA-PQP
			Chloroquine-sulfadoxine-pyrimethamine	51	41.2%	
			Artesunate-sulfadoxine-pyrimethamine	39	46.2%	
			DHA-PQP	38	15.8%	
Ratcliff et al. [62]	Adults and children ≥10 kg (Indonesia)	42 d	AL	141	57%	<0.001
			DHA-PQP	147	14%	

a Relapse rate.

b 2.7 mg/kg on day 1, 1.3 mg/kg/d for a further 4 d.

c Subsequent appearance of malaria.

d Parasitological failure at 42 d: AL 54.5%, chloroquine-sulfadoxine-pyrimethamine 65.3%, artesunate-sulfadoxine-pyrimethamine 48.7%, DHA-PQP 27.8% (*p* = 0.001 versus AL).

The longest follow-up data comparing AL (without primaquine) to chloroquine-primaquine derive from a study in 132 Chinese patients with vivax malaria [56]. While initial parasite clearance was significantly faster with AL than with chloroquine-primaquine (33.5 h versus 44.9 h, *p*<0.01), cumulative relapse rates 9 mo after the initial infection were significantly higher in the two AL dosing groups (84.9% and 78.8% versus 22.9% in patients treated with chloroquine-primaquine, *p*<0.01). This is as expected, since radical cure requires concomitant administration of primaquine.

Only one trial has evaluated the efficacy of AL versus chloroquine when both are administered in combination with primaquine in the treatment of *P. vivax* malaria [57]. In this study, 98 non-G6PD-deficient adult patients with vivax malaria in Thailand were randomized to AL or chloroquine, both administered with primaquine. Mean time to parasite clearance was shorter in the AL-primaquine group (41.6 h versus 55.8 h, *p*<0.01), and all but one of the 47 AL patients achieved parasitological cure by day 28 (97.4%). For the remaining patient, in whom parasitemia was detected at day 26, de novo reinfection was excluded since the patient had remained in hospital; relapse was considered the most likely cause. All patients receiving chloroquine-primaquine achieved parasitological cure. The authors concluded that AL with primaquine was as effective as chloroquine with primaquine for the treatment of vivax malaria [57]. It is clear that more studies are needed to specifically investigate the combined efficacy and potential toxicity of AL and primaquine. To date, however, there has been no indication of safety or toxicity concerns in patients treated for vivax infection with AL alone [34], [38], [57], [62] or in the single study of AL in combination with primaquine [57]. The absence of an effect of artemether on the metabolism of primaquine [74] and the absence of an inhibitory or induction effect of lumefantrine on the CYP enzymes involved in the metabolism of primaquine (i.e., CYP1A2 and CYP3A4 [75]) suggest that a drug–drug interaction between the two drugs is unlikely.

### Comparison with Other ACTs

Studies comparing the efficacy of artemisinin monotherapies to treat vivax malaria are scarce and no longer considered ethical. In the only such trial, several antimalarials were compared in a population of Thai patients. *P. vivax* relapse rates were similar for artemether (9/17 [52.9%]) and artesunate (12/19 [63.2%]) [38], [71]. Parasite clearance time and fever clearance time were shortest among patients receiving artemether or artesunate compared to other non-artemisinin-based antimalarials, with no statistically significant differences between the two artemisinin derivatives [38].

Two studies have compared parasitological cure rates in vivax infection using AL or DHA-PQP: both studies showed a higher cure rate for the latter [59], [62] (Table 2). A study of 161 infants and children with *P. vivax* malaria in Papua New Guinea reported a significantly higher day 28 parasitological failure rate with AL, artesunate-sulfadoxine-pyrimethamine, and chloroquine-sulfadoxine-pyrimethamine compared to DHA-PQP [59]. Ratcliff et al. [62] also observed a higher failure rate with AL versus DHA-PQP, this time in an Indonesian population [62].

### Prevention of De Novo Vivax Infection

Different studies have assessed the appearance of de novo vivax infections in patients treated with AL for falciparum [58], [61], [64], [66] (Table 3). van den Broek et al. [66] observed a significantly higher number of new *P. vivax* infections at 6 wk in Bangladeshi patients treated with AL (25/121 [20.7%]) for falciparum malaria than in those treated with artesunate-mefloquine (6/121 [5.0%]) or chloroquine-sulfadoxine-pyrimethamine (4/122 [3.3%]), with all vivax infections occurring late in follow-up (21–42 d) [66]. Similarly, in a cohort of 330 patients treated for uncomplicated falciparum malaria in Laos and followed for 42 d, five cases of vivax appeared in AL-treated individuals (5/110 [4.5%]) at a median of 28 d, compared to none in the 110 patients randomized to artesunate-mefloquine or chloroquine (not significant) [61]. Smithuis et al. [64], analyzing data from a population of 679 Myanmarese patients with *P. falciparum* monoinfection, observed that 194 (28.6%) had one or more episodes of *P. vivax* infection during the 63-d follow-up [64]. The highest rate of new vivax infections at day 63 was seen in the AL cohort, but since only ∼40% of AL-treated patients provided data at day 63, the results should be interpreted with caution (Table 3). Early appearance of vivax infections, in patients with either only falciparum infection or mixed falciparum/vivax infection at baseline, was similarly low until day 14, consistent with reports that all patients with vivax malaria achieve clinical recovery [38] and parasite clearance by day 7 [59] following AL treatment. In this study, the median time to *P. vivax* recurrence was 35, 49, 42, 49, and 56 d, respectively, in the AL, artesunate-amodiaquine fixed dose, artesunate-mefloquine loose tablet, artesunate-mefloquine fixed dose, and DHA-PQP groups, respectively (*p* = 0.0001) [64]. Other authors have reported mixed results when comparing new *P. vivax* infections in patients in whom AL or artesunate-mefloquine was administered for treatment of falciparum malaria at baseline [58], [61]. A recent meta-analysis confirms the superiority of both DHA-PQP and artesunate-mefloquine to AL in reducing the incidence (reappearance or new infection) of *P. vivax* over 42 d [39], but in view of the limited difference in the duration of partner drug half-lives, this may not be sustained in the long term. In areas of intense vivax transmission that have only limited use of primaquine, however, prevention of de novo vivax infections may be achieved more effectively by ACTs in which there is an extended partner half-life.

**Table 3:** Appearance of new *P. vivax* infections in patients with *P. falciparum* monoinfection at the time of treatment.

Study	Population (Location)	Follow-Up	Treatment	Sample Size	New Vivax Infection	*p*-Value
Hutagulung et al. [58]	Adults and children >10 kg (Thailand)	42 d	AL	225	40.0%	<0.001
			Artesunate-mefloquine	227	12.7%	
van den Broek et al. [66]	Adults and children ≥1 y (Bangladesh)	42 d	AL	121	20.7%	<0.001
			Chloroquine-sulfadoxine-pyrimethamine	122	3.3%	
			Artesunate-mefloquine	121	5.0%	
Smithuis et al. [64]	Adults and children >6 mo and >5 kg (Myanmar)	63 d	AL	137	35.8%	<0.001
			Artesunate-amodiaquine	129	29.5%	n.s.
			Artesunate-mefloquine (fixed dose)	148	31%	Reference group
			Artesunate-mefloquine (loose tablet)	130	32.3%	0.0001
			DHA-PQP	135	34%	n.s.
Mayxay et al. [61]	Adults and children ≥1 y (Laos)	42 d	AL	110	1.5%	n.s.
			Chloroquine-sulfadoxine-pyrimethamine	110	0	
			Artesunate-mefloquine	110	0	

n.s., not significant.

## Gametocidal Effect

Unlike in *P. falciparum*, where gametocytogenesis is delayed until the appearance of clinical symptoms, *P. vivax* generates gametocytes at an early stage of the infection, often preceding any symptomatology. This explains the high rate of gametocyte carriage (∼60%) often present in vivax infections [62], [71], [76]. As a result, vivax can be transmitted to other individuals before patients have started any treatment, a circumstance which is further aggravated by the fact that *P. vivax* achieves effective transmission at low blood densities.

In *P. falciparum* infections, most antimalarial agents other than ACTs have little or no effect on gametocyte development [77]–[79]. However, all antimalarial agents are considered effective against both the asexual and sexual stages of *P. vivax* malaria [71]. Few prospective studies, however, have compared the gametocytocidal activity of different therapies against vivax, and no adequate in vitro models exist. The limited data available, however, suggest that artemisinin derivatives such as AL clear *P. vivax* gametocytes rapidly [62], [71], but the overall effect on gametocyte carriage is less marked than in falciparum infections because of the proportionally higher individual burden of gametocytes that the drug has to deal with. One prospective study of 349 adults with *P. vivax* malaria in Thailand, undertaken outside a malaria transmission area, compared eight different antimalarial agents [71]. In this cohort, 77 patients (22%) had vivax gametocytemia on admission, and a further 144 (41%) developed gametocytemia after treatment. The median time to gametocyte clearance was shortest with AL (8 h), artesunate (4 h), and chloroquine-primaquine (2 h), with a markedly longer clearance time in patients receiving mefloquine (24 h), primaquine (24 h), or quinine (20 h).

In falciparum malaria, two studies have indicated that AL exerts a more potent gametocytocidal effect than DHA-PQP [80], [81], while two have suggested the converse [82], [83]; overall, the effect of these two drugs appears to be similar [39]. Only one study has compared the gametocytocidal effect of different artemisinin derivatives against vivax infection, in a cohort of 284 Indonesian patients with *P. vivax* monoinfection or mixed infection [62]. In total, 56% showed vivax gametocytes on admission. The prevalence of gametocytemia remained markedly lower with both AL and DHA-PQP during the 42-d follow-up. After day 14, vivax gametocytemia seemed to increase among AL-treated patients but declined again by day 42, while for DHA-PQP-treated patients, gametocytes did not reappear until day 28 and then began a modest growth towards the end of follow-up (the gametocyte carriage rate was 24.6 and 3.7 per 1,000 patient-weeks to day 42 for AL and DHA-PQP, respectively, *p*<0.001). Assuming that gametocytes show a similar sensitivity to all artemisinin derivatives, these findings would be consistent with the hypothesis that the effect of ACTs on vivax gametocytogenesis is influenced by the half-life of the partner drug, capable of inhibiting relapses (and hence new gametocytes) or new infections, but with possibly no direct effect on existing gametocytes.

To our knowledge, no data are available regarding the specific impact of AL on the viability of gametocytes, an essential component for malaria transmission, which is, indeed, more critical than the absolute presence or absence of gametocytes.

## Conclusions

The large burden and wide geographical distribution of *P. vivax*, and its clear recognition as a non-benign infection, calls for a paradigm change in the way we consider this infection. Effective treatment should be used rationally and rapidly, and although vivax may still be sensitive in many areas of the world to chloroquine, we need to acknowledge the new role that artemisinin derivatives, in combination with primaquine, will have to play in the short term for its control. AL is now widely deployed for the treatment of falciparum malaria, based on extensive evidence of consistently high efficacy and an extensive, convincing safety data base. Clinical trials in vivax malaria are scarcer. The available data show that AL offers good efficacy against the blood stages of *P. vivax* infection, providing rapid clearance of both parasites [38], [56], [57] and fever [56], [57]. Although ACTs such as AL, in which the partner drug has a relatively short half-life, are less vulnerable to emergence of resistant parasites, they are also associated with a shorter time to vivax recurrence. This issue could be overcome by co-administration of a full radical cure using primaquine at its standard dosage [57], although more trials are required. As with other treatments for vivax malaria, primaquine therapy should be administered in combination with AL when used to treat *P. vivax* infections, preferably after ascertainment of G6PD status. In areas of high transmission, or when G6PD deficiency cannot be easily excluded and primaquine use is erratic, other ACT combinations in which the half-life of the partner drug is longer (e.g., DHA-PQP) may be more efficacious to prevent relapses. However, the use of AL remains a valid alternative, and a pragmatic choice. Finally, AL and other artemisinin derivatives quickly clear *P. vivax* gametocytes [71], [82], but the benefit in terms of reducing transmission rate is muted by the high rate of gametocyte carriage that is typical in vivax infections [62], [71], [76].

A unified treatment strategy for the asexual forms of both falciparum and vivax infections would offer important logistical advantages. However, the incomplete nature of current data on the efficacy of AL, and of ACTs in general, in the treatment of *P. vivax* monoinfections and/or the prevention of *P. vivax* relapses or new infections compels the malaria community to assess urgently the efficacy, cost-effectiveness, safety, and toxicity of these drugs on their own and in association with effective antihypnozoite treatment.

**Key Learning Points**

- The underestimation of *P. vivax*'s real burden and potential to cause severe disease, and the identification of increasing parasite resistance in many areas of the world to chloroquine, the mainstay of its treatment, compel the malaria community to actively search for new and effective treatment strategies.
- A unified treatment strategy for both falciparum and vivax infections using ACTs that have already been deployed in many malaria-endemic areas of the world would offer important logistical and cost advantages, especially in areas of high chloroquine resistance or where parasitological diagnosis remains challenging.
- The efficacy of AL against the blood stages of *P. vivax* seems clear, showing rapid parasite and fever clearance.
- The relatively short half-life of lumefantrine means that day 28 parasitological cure rates are lower for AL than for other ACTs. This probably implies a shorter time to spontaneous relapse caused by maturation of dormant hypnozoites, and the possibility of an earlier susceptibility to new infections, rather than a genuine difference in efficacy against the blood stages of vivax infection.
- Differences between AL and other ACTs are likely to be restricted to variations in different partner drug half-lives, which may be of limited clinical relevance especially if these drugs are given in combination with primaquine, and should be balanced against the possible risk of drug resistance, which is associated with increasingly long drug half-lives.
- AL for treatment of vivax malaria may be appropriate in chloroquine-resistant *P. vivax*, and in co-endemic areas where AL is already used against *P. falciparum*, as well as when parasitological differentiation is not routinely performed and treatment is primarily based on clinical suspicion.

**Key Papers**

- Maguire JD, Baird JK (2010) The ‘non-falciparum’ malarias: the roles of epidemiology, parasite biology, clinical syndromes, complications and diagnostic rigour in guiding therapeutic strategies. Ann Trop Med Parasitol 104: 283–301.
- Pukrittayakamee S, Chantra A, Simpson JA, Vanijanonta S, Clemens R, et al. (2000) Therapeutic responses to different antimalarial drugs in vivax malaria. Antimicrob Agents Chemother 44: 1680–1685.
- Ratcliff A, Siswantoro H, Kenangalem E, Maristela R, Wuwung RM, et al. (2007) Two fixed-dose artemisinin combinations for drug-resistant falciparum and vivax malaria in Papua, Indonesia: an open-label randomised comparison. Lancet 369: 757–765.
- Smithuis F, Kyaw MK, Phe O, Win T, Aung PP, et al. (2010) Effectiveness of five artemisinin combination regimens with or without primaquine in uncomplicated falciparum malaria: an open-label randomised trial. Lancet Infect Dis 10: 673–681.
- Douglas NM, Anstey NM, Angus BJ, Nosten F, Price RN (2010) Artemisinin combination therapy for vivax malaria. Lancet 10: 405–416.
